# Rare Case of Ludwig’s Angina in a Child

**DOI:** 10.7759/cureus.40446

**Published:** 2023-06-15

**Authors:** Shikha Kakkat, Sham Lohiya, Yarraiahgari Maheswara, Jayant D Vagha, Amar Taksande, Revat J Meshram

**Affiliations:** 1 Pediatrics, Jawaharlal Nehru Medical College, Datta Meghe Institute of Higher Education and Research, Wardha, IND

**Keywords:** antibiotics, submandibular, parotid swelling, neck, ludwig’s angina

## Abstract

The parents of an 11-month-old infant presented her to the hospital due to her persistent fever and the presence of a swollen neck over a period of four days. Inflammation and discomfort were observed on both sides of her neck, particularly around the parotid glands. Notably, a localized collection of pus was identified beneath her left jaw while both sides of her jaw exhibited swelling, albeit with less sensitivity. A neck scan revealed an infection in the jaw region, accompanied by swelling in the facial skin and underlying tissue. The diagnosis rendered was Ludwig's angina, for which the prescribed course of treatment involved administering intravenous antibiotics such as amikacin, vancomycin, and Meropenem. The patient exhibited improvement after the treatment and was subsequently discharged from the hospital.

## Introduction

Ludwig's angina is an acute inflammatory condition characterized by cellulitis of the floor of the mouth. It is commonly triggered by oral sepsis. Ludwig's angina can lead to substantial edema, distortion, and airway obstruction, making it a potentially life-threatening disease. In the past, before the introduction of antibiotics, the mortality rate for Ludwig's angina exceeded 50% [1]. The swelling caused by the edematous tissues of the mouth floor pushes the tongue upward, resulting in difficulties with swallowing and breathing. Moreover, the edema may extend to the larynx.

Ludwig's angina primarily originates from dental sources [2]. Symptoms include painful swelling of the neck, toothache, difficulty swallowing, pain during swallowing, shortness of breath, fever, and general discomfort. Additional symptoms involve increased effort required for breathing, stridor, spasms of the jaw muscles leading to a tightly closed mouth, and excessive salivation. Physical examination reveals a firm and tender swelling in the submental and anterior neck regions without any fluid fluctuation. Various aerobic and anaerobic microorganisms and occasionally fungi have been implicated in the development of Ludwig's Angina. These microorganisms include oral flora such as streptococci and staphylococci [3]. Ensuring the security and patency of the airway is of utmost importance. Treatment options include the administration of broad-spectrum antibiotics and, in certain cases, drainage of the swelling [4].

There are specific risk factors associated with Ludwig's angina. Poor oral hygiene, dental infections, dental procedures, mouth or facial trauma, immunosuppression, and underlying systemic diseases, such as diabetes, can increase the susceptibility to developing Ludwig's angina. Regarding mortality and fatality, before the availability of antibiotics, Ludwig's angina had a mortality rate exceeding 50% [1]. This underscores the severity of the condition. The potential for airway obstruction due to edema and distortion, coupled with the associated difficulties in swallowing and breathing, can greatly compromise the patient's respiratory function. Prompt intervention is crucial to prevent fatal outcomes.

Modern medical advancements, including antibiotics and appropriate management strategies, have significantly improved the prognosis of Ludwig's angina. However, delayed or inadequate treatment can still lead to severe complications and potentially fatal consequences. Therefore, timely diagnosis, appropriate antibiotic therapy, and ensuring airway security remain essential in minimizing the risk of mortality associated with this condition.

## Case presentation

An 11-month-old female infant was presented by her parents at the hospital, reporting swelling in the neck region that had been present for four days. The onset of the swelling was gradual, and its progression was continuous. According to the parents, the patient also experienced episodes of fever and increased effort in breathing. The swelling initially appeared below the left ear and gradually enlarged, affecting both sides of the jaw area.

Upon examination, the patient had a body temperature of 100.4 degrees Fahrenheit, a pulse rate of 122 beats per minute, and a respiratory rate of 62 breaths per minute. During a neck examination, a purulent point was discovered below the left mandible, along with diffuse swelling in the submandibular region on both sides. The consistency of the swelling was firm, and mild tenderness was observed. Examination of the ears, nose, and oral cavity revealed no abnormalities.

**Figure 1:**
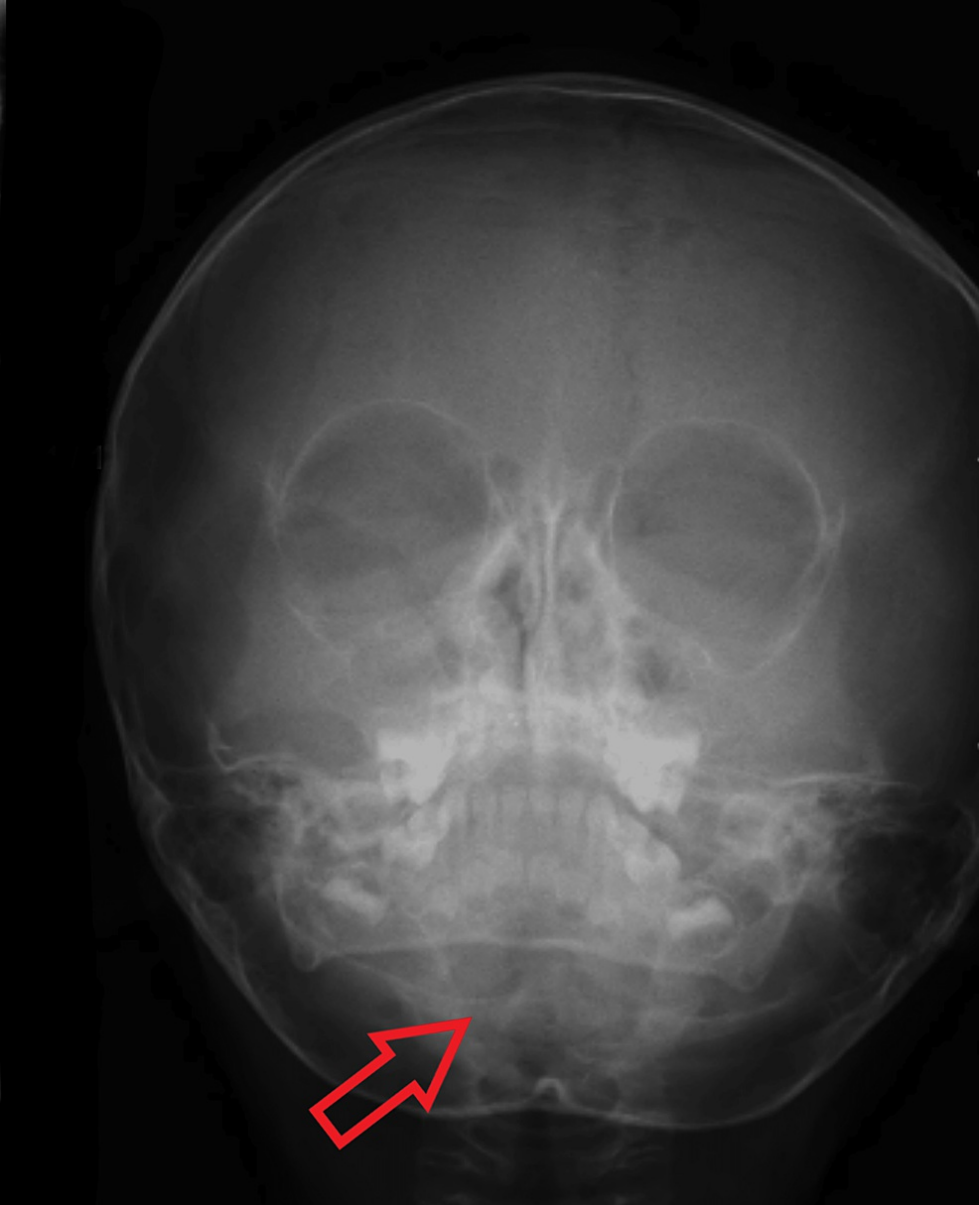
Red arrow indicates Caldwell’s perspective on Ludwig’s angina in an 11-month-old female infant

A neck ultrasound was performed due to cellulitis in the bilateral mandibular region and inflammatory edema in the facial skin and subcutaneous tissue (Figure 1). Subsequently, the patient developed a grunting sound and respiratory distress, leading to the decision to intubate her. Antibiotics such as intravenous meropenem, vancomycin, and amikacin were administered. Pus was drained from the affected area, and a culture was conducted, which revealed the presence of oxacillin-sensitive, coagulase-positive staphylococci that were sensitive to vancomycin. The swelling subsided as treatment progressed, oxygen support gradually reduced, and the patient was extubated.

The patient's vital signs remained stable, her respiratory distress improved, and her overall clinical condition improved. Her symptoms continued to improve, and she could easily consume oral feeds. Consequently, she was discharged from the hospital in a stable condition.

## Discussion

The presented case describes Ludwig's angina in an 11-month-old female infant, a potentially life-threatening condition characterized by severe cellulitis and edema involving the submandibular and sublingual spaces. This case is particularly interesting due to the patient's young age and the subsequent successful management of the condition.

Ludwig's angina typically occurs due to odontogenic infections, with the most common causative organisms being Streptococcus and Staphylococcus species [5,6]. In this case, a culture revealed the presence of oxacillin-sensitive, coagulase-positive Staphylococci, highlighting the importance of microbiological investigations for appropriate antibiotic selection.

Morbidity and mortality rates associated with Ludwig's angina have historically been high, especially in delayed diagnosis or inadequate treatment [7,8]. However, advancements in diagnostic techniques, improved access to healthcare, and the availability of potent antibiotics have significantly reduced the mortality rate associated with this condition [9]. Promptly recognizing clinical signs and symptoms and early intervention can greatly improve patient outcomes.

The patient presented with progressive swelling, fever, and respiratory distress in this case. The decision to perform a neck ultrasound was crucial in confirming the diagnosis and guiding appropriate management. Intubation was necessary due to respiratory distress and the grunting sound, indicating compromised airway patency. The administration of intravenous antibiotics, including Meropenem, Vancomycin, and Amikacin, was initiated promptly. The culture results guided the selection of appropriate antibiotics, targeting the identified staphylococcal infection. Drainage of the purulent material also played a crucial role in reducing the swelling and facilitating the patient's recovery [10].

Notably, the patient's clinical condition improved steadily, as evidenced by the reduction in swelling, resolution of respiratory distress, and ability to consume oral feeds. These positive outcomes can be attributed to the early diagnosis, prompt initiation of appropriate antibiotic therapy, and timely drainage of the abscess. This case's uniqueness lies in the patient's age, as Ludwig's angina is relatively rare in infants. The management of Ludwig's angina in this age group requires careful attention to airway management and antibiotic selection, considering the limited tolerability of certain drugs in young children.

## Conclusions

Ludwig's angina necessitates prompt recognition and appropriate treatment due to the potential development of severe respiratory distress, airway obstruction, and even fatal outcomes in the advanced stages of the condition. The early initiation of antibiotic therapy assumes a crucial role in effectively managing this condition. In cases that have progressed significantly, ensuring the unobstructed airflow through the airway and performing pus drainage are indispensable components of the treatment approach. In conclusion, although Ludwig's angina carries notable risks in terms of morbidity and mortality, early recognition, administration of suitable antibiotics, and timely interventions can lead to positive outcomes. This successful management of Ludwig's angina in an 11-month-old female infant highlights the potential for favorable results even in young children. The uniqueness of this case lies in the patient's age, underscoring the significance of tailored management strategies for infants diagnosed with Ludwig's angina.
